# γδTCR regulates production of interleukin-27 by neutrophils and attenuates inflammatory arthritis

**DOI:** 10.1038/s41598-018-25988-3

**Published:** 2018-05-15

**Authors:** Laura Bouchareychas, Eva M. Grössinger, Mincheol Kang, Iannis E. Adamopoulos

**Affiliations:** 1 Division of Rheumatology, Allergy and Clinical Immunology, University of California, Davis, USA; 2 Institute for Pediatric Regenerative Medicine, Shriners Hospitals for Children Northern California, Sacramento, USA

## Abstract

γδ T cells have been implicated in inflammatory diseases as an important link between the innate and adaptive immune responses, however, their role in inflammatory arthritis remain unclear. To define the contribution of γδ T cells in the pathogenesis of inflammatory arthritis, we performed gene transfer of IL-23 in B10.RIII mice to establish joint inflammation in the presence or absence of γδ T cells. We demonstrated that γδ T cell blockade has a protective effect on arthritis incidence and severity by preventing neutrophil accumulation in the blood, spleen and bone marrow as well as by reducing neutrophil infiltration into the joints. Furthermore, our data demonstrate that absence of γδ T cells was associated with an increase of IL-27 levels produced by neutrophils and dendritic cells, and systemic IL-27 expression also prevents IL-23-induced inflammatory arthritis and limits neutrophil expansion. Collectively our findings reveal an immunomodulatory effect of γδ T cells on neutrophils associated with IL-27 synthesis and secretion and indicate a novel link between IL-27 and the modulation of γδ T cells and neutrophils that can be targeted in the treatment of inflammatory arthritis.

## Introduction

Gamma delta (γδ) T cells are a minor population of T cells that express the T-cell receptor γδ chains, accounting for less than 5% of the total T cells in the peripheral blood of mice and humans and are more commonly localized in mucosal tissues, such as the gut, skin and lung^1,2^. These cells exhibit different functional activity with an adaptive potential and an innate-like capacity to respond to pro-inflammatory cytokines in the absence of further antigens^3^. γδ T cells can produce high levels of interferon-γ (IFN-γ) and tumor necrosis factor (TNF), Interleukin 17 (IL-17) and large amounts of chemokines reflecting their role in the effector phase of immune response^4^. In this regard, γδ T cells may participate in the early stages of inflammation in synchrony with innate immune cells.

γδ T cells are known to have a strong clinical association with many autoimmune diseases, such as rheumatoid arthritis (RA) but their function in disease activity is not clearly understood. Significantly higher levels of γδ T cells are found in RA patients^5,6^ associated with enhanced IL-17 secretion^7^ and hyperplasia of the synovial tissue and progressive destruction of joint structure. The role of γδ T cells has been documented in the collagen-induced arthritis (CIA) animal model of experimental arthritis where γδ T cells depletion prior to disease induction delayed both the onset and severity of the disease. In contrast, depletion of γδ T cells in established arthritic mice accelerated cellular infiltration into the joint and induced bone erosion^8^. These data suggest that γδ T cells might exhibit different functions depending on other effector cells present in the inflammatory environment of the joint.

A strong link between the proinflammatory IL-23/IL-17 axis and γδ T cells lineage has been established. IL-23 is produced by innate immune cells and is an essential mediator of joint inflammation and is critical for induction of arthritis, osteoclast formation, and maintenance of bone mass^9,10^. γδ T cells express constitutively high amounts of IL-23 receptor (IL-23R) that drives their expansion and therefore their secretion of IL-17^11^.

Several studies demonstrated that γδ T cells are a predominant source of IL-17 in the swollen joints of mice with CIA^12,13^ suggesting that cytokine process may drives the pathogenic effect of γδ T cells. The dependence of arthritis initiation on IL-17 alone seems highly unlikely as we have shown that IL-17 alone is not capable of inducing arthritis *in vivo*^9^ although we and others have shown that IL-17 can very well exacerbate established arthritis^14,15^.

Another potent mechanism that may modulate γδ T cells function is their ability to interact with other innate immune cells. In models of bacterial infection, γδ T cells were found to control neutrophil infiltration^16,17^. This notion is also consistent with a recent study reporting γδ T cells and neutrophils conspiration to promote breast cancer metastasis^18^. In inflammatory conditions such as RA, neutrophils play a role in the persistence of inflammation and progression of joint damage. Increased numbers of neutrophils have been found in the synovial fluid of patients with RA^19,20^. In the arthritis animal model induced by anti-type II collagen (CII) antibodies and lipopolysaccharide (LPS) injection, neutrophils are the major population of infiltrating cells in the joint space and neutrophil depletion using mouse antibody (mAb) against Gr-1 *in vivo* suppresses the development of arthritis^21^. Furthermore, neutrophil depletion renders mice resistant to K/B × N serum-induced joint inflammation^22^. Kim *et al*. showed that neutrophils were crucial for arthritis generation and chemokine production in the K/BxN mouse model^23^. The prominent role of neutrophils and γδ T cells in inflammatory arthritis, with regard to their localization, cytokine production and interaction with other immune cells that influence pathogenesis merits further investigation.

In this study, we describe the involvement of γδ T cells to the pathogenesis of IL-23-induced arthritis mice model and further evaluated the impact on myeloid cells. We found that γδ T cell blockade prior IL-23 MC injection significantly reduced both incidence and disease severity score by suppressing neutrophil expansion and increasing IL-27 levels. Furthermore, IL-27 gene transfer prior IL-23 MC injection inhibits arthritis development and both neutrophils and γδ T cell expansion. Collectively our data describe a novel interplay between γδ T cells and neutrophil secretion of IL-27, which negatively regulates inflammatory arthritis.

## Results

### Protective effect of γδ T cell blockade in IL-23-induced arthritis

**Figure 1:**
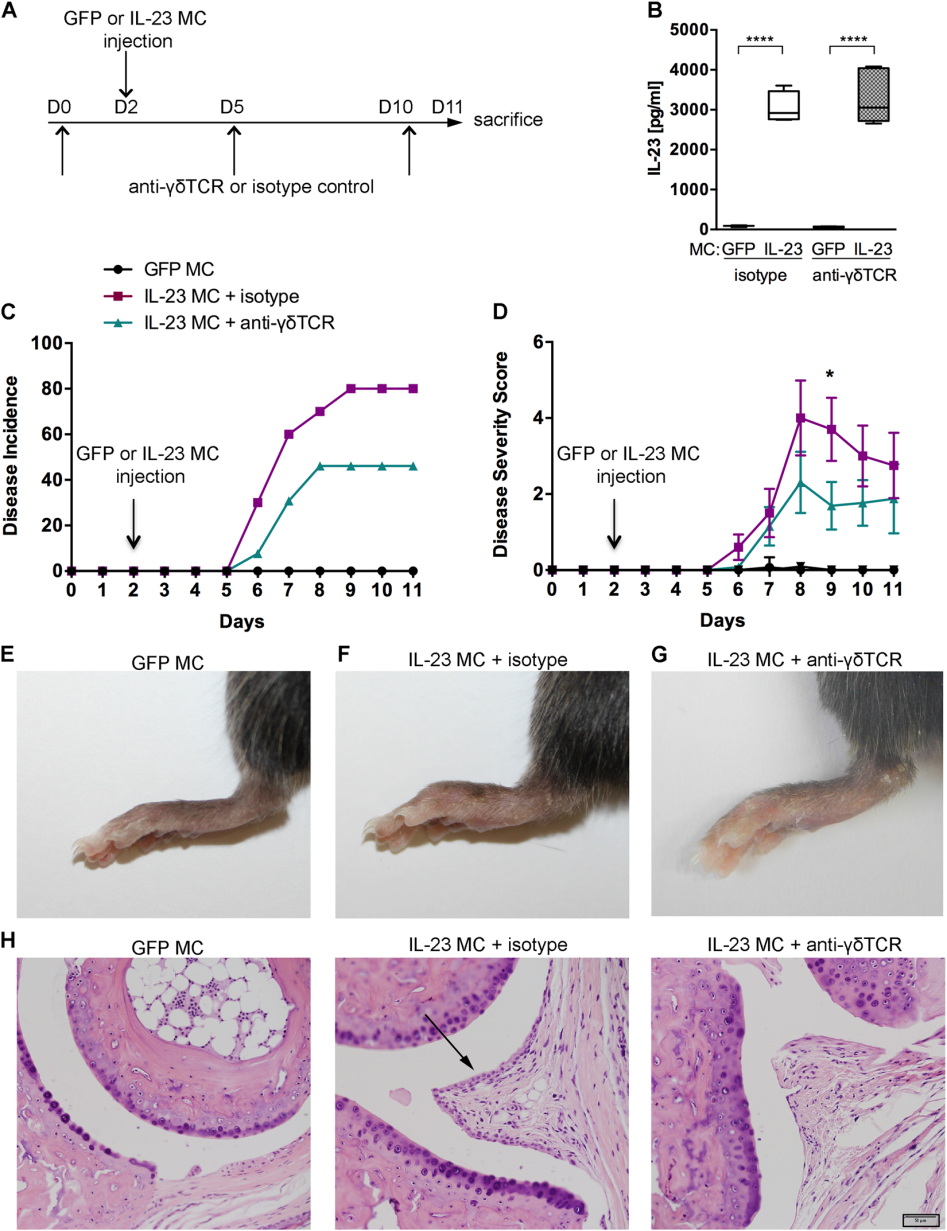
Decrease of IL-23-induced arthritis in anti-γδ TCR mAb treated mice. (**A**) Schematic illustration of the experimental protocols. B10.RIII mice at the age of 10–12 weeks were treated on days 0, 5 and 10 with anti-γδ TCR or isotype control mAb prior to GFP or IL-23 MC injection at day 2 (n = 10–13 per group). (**B**) Serum IL-23 levels by ELISA of each group (n = 4–5 per group). Median, interquartile minimum, and maximum range is depicted by box plots, ****p < 0.0001 by one-way ANOVA with Sidak’s multiple comparisons test. (**C**) Time course of disease incidence and (**D**) severity score of arthritis in mice injected with GFP or IL-23 MC and treated with anti-γδ TCR or isotype mAb (n = 10–14 per group). *p < 0.05 by using two-tailed Student’s t-test. Representative pictures showing the hind paws of B10.RIII mice injected with GFP MC (**E**) or IL-23 MC + isotype (**F**) or IL-23 MC + anti-γδ TCR (**G**). (**H**) Representative H&E stained sections of day-11 metatarsophalangeal joint from GFP MC and IL-23 MC injected mice treated with isotype (middle column) or γδ TCR mAb (right column) are shown (20× objective). The black arrow indicates synovial hyperplasia. Scale bars, 50 μm. Data are representative of three independent experiments. All data are shown as mean ± SEM.

To analyze the functional role of γδ T cells in IL-23-induced arthritis, we performed IL-23 or GFP control *in vivo* gene transfer in B10.RIII mice as previously described^24^ to induce inflammatory arthritis in the presence or absence of γδ T cells (Fig. 1A). IL-23 MC injected mice revealed a significant elevation of serum IL-23 whereas GFP MC injected mice did not have detectable levels of IL-23 (Fig. 1B). Blockade of γδ T cells by anti-γδ TCR mAb was performed 2 days prior gene transfer and analyzed by flow cytometry in the spleen and draining lymph nodes. Our data showed that antibody blockade at the selected dose was comparable with TCRδ^−/−^ deficient mice (Supplemental Fig. 1A). Administration of the anti-γδ TCR or isotype mAb did not affect myeloid populations in the blood (Supplemental Fig. 1B,C), spleen (Supplemental Fig. 1D) or bone marrow, as confirmed by flow cytometry (Supplemental Fig. 1E). Our results show that γδ T cell blockade prior to IL-23 gene transfer caused a marked decrease (46.15%) in disease incidence compared to controls (80%) at day 11 post-gene transfer (Fig. 1C). γδ T cell blockade also resulted in a significant decrease of the disease severity score as compared to control mice (Fig. 1D) as shown by reduced paw swelling in the γδ T cells depleted group in our arthritis model (Fig. 1E–G). Histologic assessment of the ankle joints revealed a marked synovial hyperplasia in mice injected with IL-23 MC, which is reduced in anti-γδ TCR mAb-treated mice. Representative sections of the average disease score (mild inflammation) are shown (Fig. 1H). These observations suggest that γδ T cells play a pathogenic role in supporting the development of arthritis in IL-23 gene transfer model of inflammatory arthritis. Next, we examined the potential cellular mechanisms that are responsible for the protective effect of γδ T cell blockade.

### γδ T cells regulate the expansion and recruitment of neutrophils

**Figure 2:**
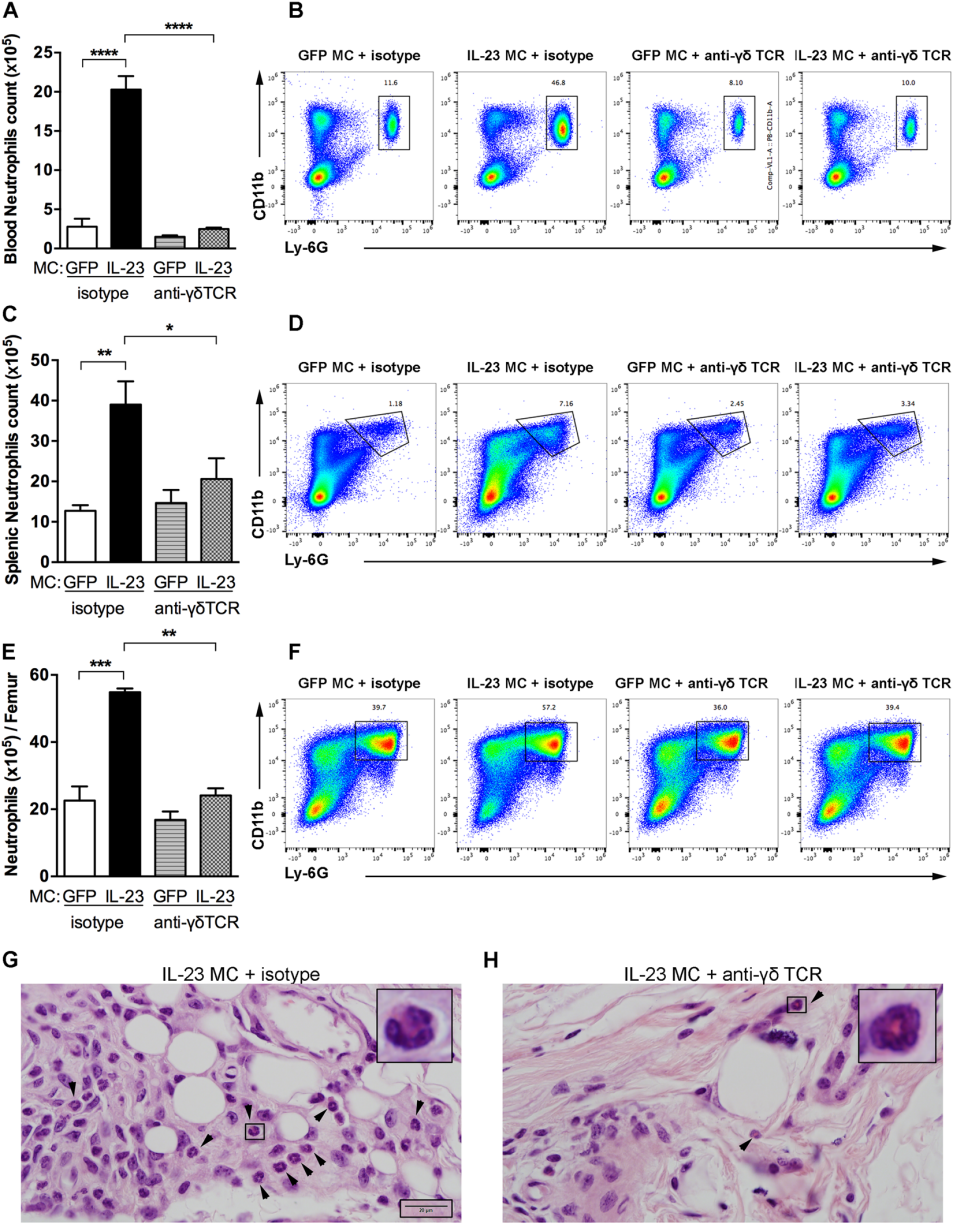
Anti-γδ TCR treatment inhibits the expansion of neutrophils. (**A**) Quantification of CD11b^+^ Ly-6G^+^ blood neutrophils in B10.RIII mice injected with GFP or IL-23 MC and treated with anti-γδ TCR or isotype mAb determined by flow cytometry. (**B**) Representative flow cytometry plots showing the gating strategy for evaluating the numbers of blood neutrophils. (**C**) Splenic CD11b^+^ Ly-6G^+^ neutrophil counts in B10.RIII mice injected with GFP or IL-23 MC and treated with anti-γδ TCR or isotype mAb determined by flow cytometry. (**D**) Gating strategy for evaluating the percentage and absolute numbers of neutrophils in mouse spleen. (**E**) Absolute numbers of CD11b^+^ Ly-6G^+^ neutrophils per femur. (**F**) Representative flow cytometry plots showing the gating strategy. Numbers depict the percentage of CD45^+^ leukocytes. (n = 4–5 per group). (**G**,**H**) Representative haematoxylin and eosin-stained sections of day-11 ankles histopathology from mice receiving isotype control or anti-γδ TCR mAb showing neutrophils in joint capsule imaged with 100 × oil-immersion objective lense. Arrows indicate polymorphonuclear neutrophils. Scale bars, 20 μm. Data were obtained from 3 independent experiments (n = 4–5 per group). All data are shown as mean ± SEM. *p < 0.05, **p < 0.01, ***p < 0.001, ****p < 0.0001. Statistical analysis was performed using one-way ANOVA with Sidak’s multiple comparisons test.

We previously showed that systemic IL-23 exposure induced myelopoiesis in the bone marrow and the spleen^9,24^. To investigate whether γδ T cells affect IL-23-induced myelopoiesis, myeloid cells were analyzed in B10.RIII mice injected with GFP or IL-23 MC and treated with anti-γδ TCR or isotype mAb. We found that IL-23 MC injection increases neutrophil populations in the blood compared to GFP MC. Interestingly, blood CD11b^+^ Ly-6G^+^ neutrophils were significantly reduced in mice injected with IL-23 MC and treated with anti-γδ TCR mAb compared to control mice as shown in the representative FACS-plots and total counts (Fig. 2A,B**)**. Analysis of splenic neutrophils also showed a significant increase in IL-23 injected mice compared to GFP MC, which again was inhibited by γδ T cell blockade (Fig. 2C,D**)**. Splenic CD11b^+^ CD64^+^ macrophages as well as CD11c^hi^ MHCII^+^ dendritic cells count remained unchanged (data not shown). Similarly, analysis of bone marrow isolated cells revealed an increase of neutrophils in IL-23 MC injected mice compared to control mice which was again significantly reduced in anti-γδ TCR mAb treated mice as shown in the representative FACS-plots and total counts (Fig. 2E,F**)**. To assess whether γδ T cells promote neutrophil migration into the joint, neutrophils were visualized by H&E staining within the joint capsule. We found that IL-23 MC gene transfer induced a marked neutrophil infiltration into the joint (Fig. 2G**)**, which was reduced by γδ T cell blockade (Fig. 2H**)**. Neutrophil elevation was not detected in the synovium of mice treated with GFP MC (data not shown). These data suggest that γδ T cells are able to modulate the IL-23-induced neutrophil expansion. We next investigated any possible molecular changes that might be affected by γδ T cell blockade.

### γδ T cell deficiency increases IL-27 levels

**Figure 3:**
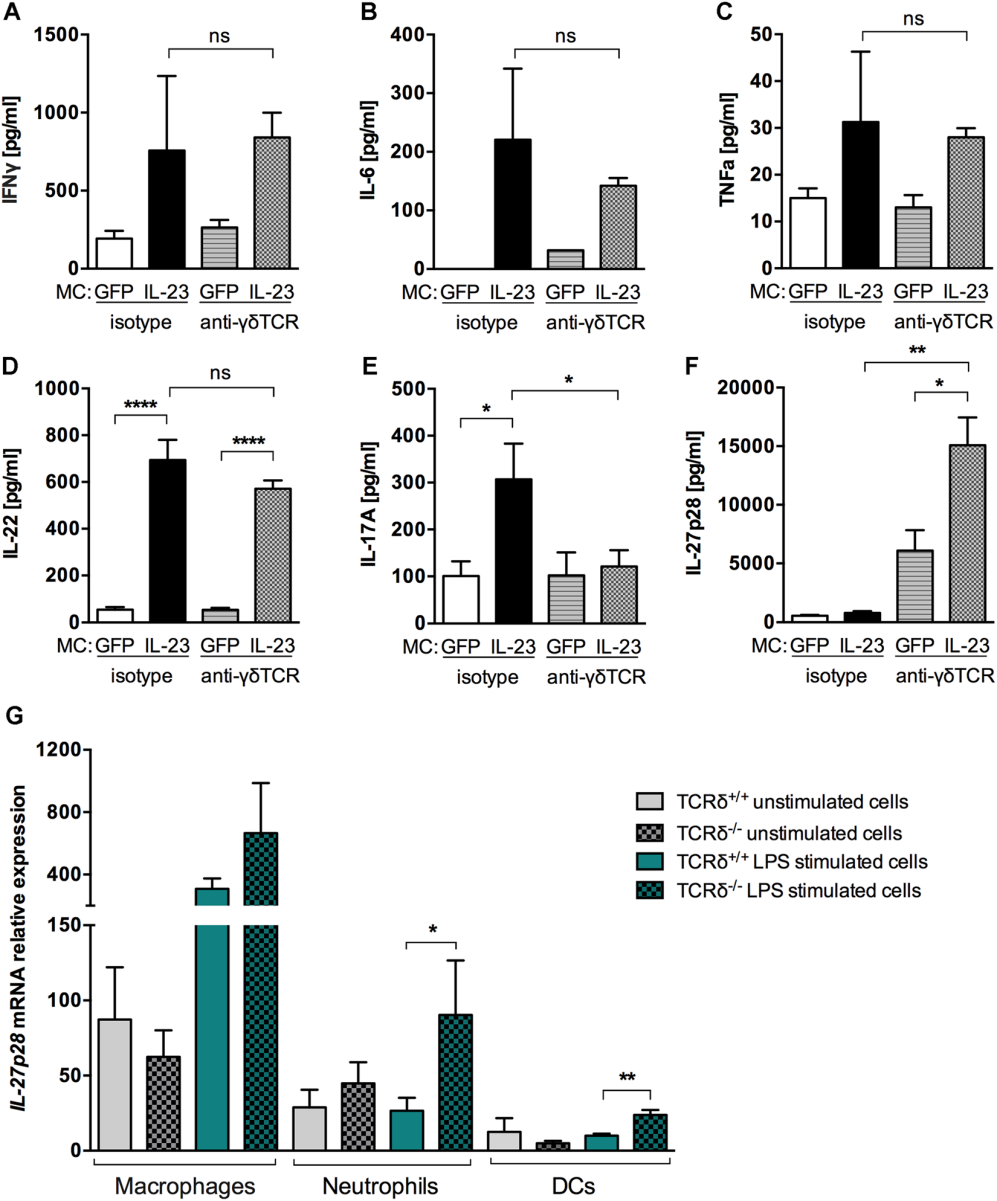
γδ T cell blockade increases IL-27 levels. (**A**–**F**) Cytokine levels in serum of B10.RIII mice injected with GFP or IL-23 MC and treated with anti-γδ TCR or isotype mAb on day 11 (n = 4–5 per group). (**G**) qRT–PCR expression analysis of IL-27p28 mRNA expression in sorted macrophages, neutrophils and dendritic cells (DCs) isolated from TCRδ^+/+^ or TCRδ^−/−^ mice after 5 hours of LPS stimulation. Data are representative of three independent experiments. All data are shown as mean ± SEM. ns, not significant (*p* > *0*.*05*) *p < 0.05, **p < 0.01, ***p < 0.001, ****p < 0.0001 as determined by using two-tailed Student’s t-test.

IL-23 MC gene transfer induced the expression of pro-inflammatory cytokines as previously shown^9^, however treatment with UC7-13D5 mAb did not have a profound effect on the expression of IFNγ, TNF, IL-6, and IL-22 (Fig. 3A–D), with the exception of a marked decrease in IL-17A serum concentration (60.83%) (Fig. 3E).

In contrast, IL-27p28 serum levels, which were not increased with IL-23 MC injection in isotype mAb treated mice, were markedly increased in the absence of γδ T cells (Fig. 3F). Therefore, we next identified the IL-27p28 producing cells by challenging splenocytes isolated from TCRδ^+/+^ or TCRδ^−/−^ mice with LPS, a known inducer of IL-27 production^25^. Then, macrophages, dendritic cells and neutrophils were sorted based on gated strategy used in Supplemental Fig. 2A and we tested their ability to produce IL-27p28. Our data show that IL-27p28 mRNA expression was mainly produced by activated macrophages and neutrophils (Fig. 3G). In addition, we demonstrated that neutrophils and dendritic cells isolated from TCRδ^−/−^ mice express more IL-27p28 mRNA compared to TCRδ^+/+^ isolated cells as analyzed by qPCR. Taken together our results indicate that γδ T cells have an important regulatory effect on IL-27 synthesis. Next, we investigated whether increased IL-27 production affect the development of IL-23-induced arthritis.

### IL-27 gene transfer inhibits IL-23-induced arthritis by negative regulation of neutrophil motility and γδ T cells population

**Figure 4:**
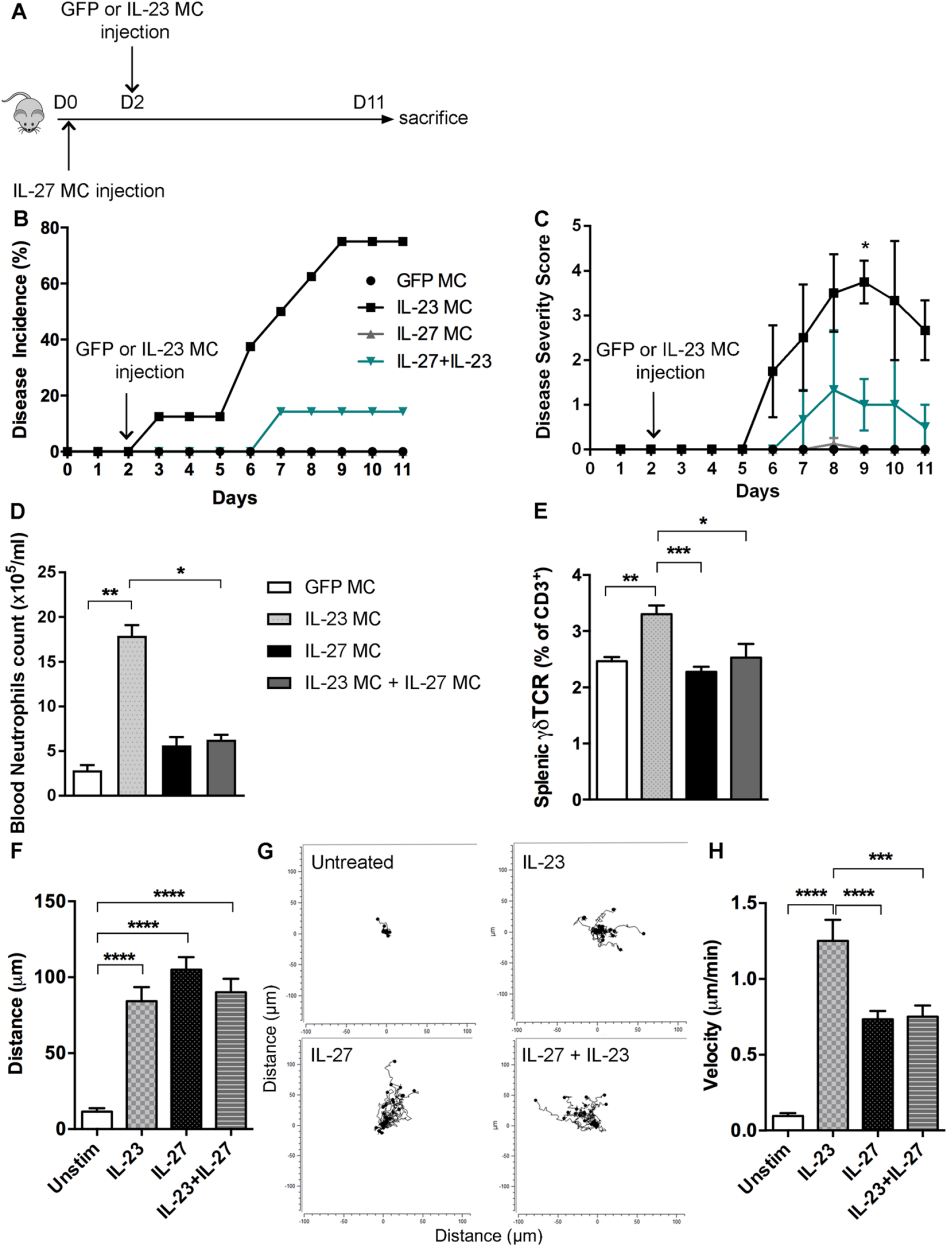
IL-27 inhibitory effect on the development of IL-23-induced arthritis. (**A**) Schematic illustration of the experimental protocols. B10.RIII mice at the age of 10–12 weeks were injected on days 0 with IL-27 MC prior GFP or IL-23 MC injection at day 2. (**B**) Time course of disease incidence and (**C**) severity score of arthritis in B10.RIII mice after GFP MC, IL-23 MC, IL-27 MC, or IL-23 + IL-27 MC injection (n = 7–10 per group). Data are representative of three independent experiments. All data are shown as mean ± SEM. *p < 0.05, as determined by using two-tailed Student’s t-test. (**D**) Quantification of CD11b^+^ Ly-6G^+^ blood neutrophils in mice injected with GFP MC, IL-23 MC, IL-27 MC or IL-23 + IL-27 MC. (**E**) Percentage of splenic γδ T cells analyzed by flow cytometry of the isolated splenocytes of each group. (**F**) Analysis of neutrophil migratory distance (in μm) under IL-23, IL-27 or IL-23 + IL-27 stimulation. (**G**) Representative neutrophil trajectory plots over a 2-hour period. (**H**) Analysis of neutrophils velocity (μm/min) under IL-23, IL-27 or IL-23 + IL-27 stimulation. Data are representative of two independent experiments. All data are shown as mean ± SEM. *p < 0.05, ** *p* < 0.01, ***p < 0.001, ****p < 0.0001. Statistical analysis was performed using one-way ANOVA with Sidak’s multiple comparisons test.

To determine whether IL-27 modulates disease severity *in vivo*, IL-27 MC alone or prior IL-23 MC gene transfer was administered by hydrodynamic injection into B10.RIII mice (Fig. 4A). Quantification of serum IL-27 taken from periodic tail bleeds demonstrated that IL-27 was stably expressed for a period of at least 11 days (data not shown). We found that IL-27 MC injection is unable to induce disease development. However, IL-27 MC injection prior IL-23 MC gene transfer significantly ameliorated both disease incidence (Fig. 4B) and disease severity (Fig. 4C) in the IL-23-induced arthritis. To determine whether IL-27 MC affects myeloid cell populations, we quantified blood monocytes and neutrophils by flow cytometry. Monocyte numbers were not affected by IL-27 injections (data not shown). However, IL-23 MC injection drastically increased blood neutrophil numbers and IL-27 MC injection suppressed the IL-23-induced neutrophil expansion (Fig. 4D and Supplemental Fig. 2B). Interestingly, we identified by flow cytometric analysis an increase of γδ T cells in the spleens of IL-23 MC injected mice compared to GFP control or IL-27 MC injected mice (Fig. 4E and Supplemental Fig. 2C). Moreover, we found that IL-27 is also able to inhibit IL-23 induced splenic γδ T cell accumulation. We next assessed how IL-27 modulates neutrophil migration in chemotaxis experiments where neutrophils were exposed to a diffusion gradient of IL-27 or IL-23 or both, IL-23 and IL-27. We did not detect any changes in directed displacement in the *x* or *y* direction in the absence of chemotactic factors (untreated). Interestingly, our data revealed that IL-23 and IL-27 are both able to increase neutrophils migration (Fig. 4F,G**)**. Moreover, both IL-23 and IL-27 were able to increase the migration velocity of neutrophils. Interestingly, neutrophil stimulation with both IL-23 and IL-27 revealed a reduced migration velocity compared to IL-23 alone (Fig. 4H). Taken together, our data show that IL-27 negatively regulates IL-23-induced arthritis by decreasing the expansion and motility of neutrophils and by reducing γδ T cells during arthritis development.

## Discussion

This study provides novel molecular insights into the regulation of neutrophils by γδ T cells. We identified a pathogenic role played by γδ T cells on inflammatory arthritis in the IL-23 gene transfer model by regulating the expansion of the neutrophil population as well as IL-27 synthesis and secretion. Furthermore, we reported that IL-27 is able to suppress IL-23-induced arthritis through limiting neutrophil expansion and migration velocity as well as γδ T cell accumulation. By this mechanism, γδ T cell blockade exert a protective effect on IL-23-induced inflammatory arthritis.

Although, there is a single report that treatment with anti-γδ TCR mAb leads to downregulation of the TCR rather than γδ T-cell depletion^26^, others have shown significant effects of the treatment with anti-γδ TCR mAb on disease incidence and severity indicating at least, that the treatment with UC7-13D5 mAb leads to functional impairment of γδT cells^27–29^. Furthermore, we found consistent results between B10RIII mice that received the UC7-13D5 mAb and TCRδ^−/−^ mice.

In our study, we demonstrated an increase of IL-17 expression in IL-23 injected mice, which is reduced with anti-γδ TCR treatment. These results suggest that γδ T cells activated by IL-23 are an important source of IL-17, which could subsequently impact arthritis development. Indeed, several observations in animal models point to the importance of IL-17 in driving synovial inflammation and joint destruction^15,30^. Although γδ T cells are an important source of IL-17, we do not exclude the possibility that other cells types might secrete IL-17 in response to IL-23 gene transfer. Indeed, natural killer (NK) cells, lymphoid-tissue inducer (LTi)-like cells and neutrophils have been described in the literature as an early source of IL-17 in response to IL-23 signalling^31,32^. However, the signaling pathway (receptors and transcription factors) required for most IL-17-producing cells remain unclear.

Nevertheless, pleiotropic functions of γδ T cells have been described in the literature and our results highlight an alternative mechanism, by which γδ T cells may mediate inflammatory arthritis, by regulating neutrophils. We identified that IL-23 induces the expansion of neutrophils into the blood, spleen, bone marrow and increased neutrophil infiltration into the joint, which was significantly reduced in anti-γδ TCR mAbs treated mice. This finding points to an immunomodulatory effect of γδ T cells on neutrophils, which could subsequently impact arthritis development.

Together, these results show that γδ T cells stimulation by IL-23 enhances the production of IL-17A by both neutrophils and γδ T cells. IL-17A elevates neutrophil counts, and increases their recruitment into the joint leading to inflammatory arthritis. Indeed, critical roles for neutrophils in initiating and maintaining joint inflammatory processes have been described in experimental arthritis mouse models^33^. Once in the joint, neutrophils perpetuate their own recruitment by releasing chemotactic factors contributing to the chronicity of the disease^23,34^. In this setting, IL-27p28 is not increased suggesting that macrophage and neutrophil activation by IL-23 and IL-17 alone is not sufficient to induce IL-27p28 expression. However, we show that IL-23 stimulation in absence of γδ T cells and neutrophil activation failed to increase IL-17 secretion and decrease inflammatory arthritis. We hypothesize that γδ T cells might inhibit/downregulate IL-27p28 expression by macrophages and/or neutrophils. This would imply that targeting the factor by which γδ T cells modulate neutrophils might be a successful therapeutic strategy for inflammatory arthritis.

The mechanism of γδ T cells migration into inflammatory sites is poorly understood as well as the heterogeneity of the local γδ T cells. Both Vγ6+ and Vγ4+ γδ T cells has been shown to be recruited to the joints, but only the Vγ6+ subset efficiently produced IL-17^35^. However, the differences between the pathogenic roles of these subsets, particularly the contribution to inflammatory diseases, remain unclear. Prospective studies should further investigate specific subsets of γδ T cells able to modulate neutrophils.

In this study, we identified a molecular interplay between IL-27, γδ T cells and neutrophils suggesting that IL-27 could be the mechanism by which γδ T cell regulates neutrophil expansion and subsequently impact arthritis development. With a combination of *in vivo* and *in vitro* experiments, we demonstrate that IL-27 is significantly increased in the absence of γδ T cells indicating a regulatory role of γδ T cells on IL-27 independently of GFP or IL-23 MC injection. Our data show that IL-27p28 mRNA expression was mainly produced by macrophages and neutrophils.

Our results suggest that despite a reduction of neutrophils count in the blood, the spleen and the bone marrow upon anti-γδ TCR treatment in arthritic mice, those neutrophils are able to increase IL-27 production. Indeed, number of cells and activity of cells are not always related. Furthermore, several studies reported the presence in circulation and tissue of distinct subsets of neutrophils, characterized by the expression of different markers^36,37^. However, it remains to be shown whether γδ T cell could modulate or switch neutrophils subsets.

Moreover, we found that IL-27 is also able to inhibit IL-23 induced splenic γδ T cell accumulation showing that IL-27 acts as a reciprocal regulator of γδ T cells. In keeping with our data, F. Morandi *et al*. provided us with the first demonstration that IL-27 modulates human γδ T cell functions *in vitro*^38^. Furthermore, IL-27 has been shown to inhibit the differentiation of Th17 cells and reduces the production of IL-17 in both, human and experimental autoimmune encephalomyelitis^9,39,40^ as well as CIA model^41^. This is consistent with studies demonstrating that IL-27 reduces the development of CIA and prevents progression of articular damage^42,43^. Similar to these observations in our data overexpression of IL-27 also inhibited arthritis initiation and progression. Mechanistically this was mainly due to a suppression of the IL-23-induced neutrophil expansion and neutrophil migration velocity. Keeping with our observations, Watzlawick, R. and al. demonstrated that IL-27 treatment inhibits neutrophil accumulation in peritonitis^42^. We hypothesize that IL-27 might regulate the expression of protein implicates into the leukocyte adhesion cascade (low rolling, adhesion strengthening, and intraluminal crawling). This may be linked with decreased Mac-1 expression in human neutrophils and suppressed neutrophil adhesion as well as LPS-induced ROS production and expression of cytotoxic granule components by IL-27^44^. While several studies indicate that γδ T cells support neutrophil recruitment, we do not exclude the possibility of the inverse relationship in which neutrophils modulate γδ T cells as it has been shown in human studies suggesting a bi-directional cross talk between γδ T cells and neutrophils^43,45^. Prospective studies should further investigate the specific subset of γδ T cells able to modulate this relationship.

In conclusion, our results establish a mechanistic connection between γδ T cells and neutrophils via IL-27. IL-27 suppresses the activation of γδ T cells and neutrophil expansion, thereby contributing to the resolution of inflammation. Accordingly, limiting the activities of γδ T cells may provide important insights and new treatment avenues for autoimmune and inflammatory diseases.

## Materials and Methods

### Reagents and mice

Male B10.RIII-*H2r H2-T18b*/(71NS)SnJ (B10.RIII), C57BL/6J (TCRδ^+/+^) and δ-chain TCR^−/−^ mice (TCRδ^−/−^) mice were purchased from Jackson Laboratories (Sacramento, CA, USA). Sex- and age-matched mice at 12–14 weeks of age were used for each experiment. The University of California at Davis Institutional Animal Care and Use Committee approved all animal protocols. All experiments were performed in accordance with relevant guidelines and regulations. Serum samples were analyzed for cytokine protein levels using either ELISA kits for IL-27p28 (from R&D Systems), IL-17 and IL-23 from BioLegend in accordance with the manufacturer’s instructions or Th17 Bead-Based Multiplex Assays (Millipore). Flow cytometry antibodies were purchased from BioLegend (San Diego, USA). Splenocytes stimulation with LPS were performed in RPMI 1640 medium containing 10% FBS, 2 mM glutamine, penicillin/streptomycin (100 IU/mL) (Life Technologies). LPS (Sigma L4524 from *Escherichia Coli* 055:B5).

### Production and purification of GFP, IL-23 and IL-27 minicircle DNA and hydrodynamic delivery

Minicircle-RSV.Flag.mIL23.elasti.bpA, p2øC31-RSV-PPT-FLAG-mIL-27.Elasti.bpA and RSV.eGFP.bpA was produced as described by Chen *et al*.^46^. Briefly, a single isolated colony from a fresh plate was grown for 8 h in 2 ml Luria-Bertani broth with appropriate antibiotic. Eight hundred microliters of this culture was used to inoculate 1 L Terrific broth and grown for an additional 17 h. Overnight cultures were centrifuged at 20 °C, 4000 rpm for 20 min. The pellet was resuspended 4:1 (v/v) in fresh Luria-Bertani broth containing 1% L-arabinose. The bacteria were incubated at 32 °C with constant shaking at 250 rpm for 2 h. After adding half volume of fresh low-salt Luria-Bertani broth (pH 8.0) containing 1% L-arabinose, the incubation temperature was increased to 37 °C and the incubation continued for an additional 2 h. Episomal DNA circles were prepared from bacteria using plasmid purification kits from Endofree Qiagen Megaprep (Chatsworth, CA, USA). Hydrodynamic delivery of MC DNA using the tail vein was performed as previously described^9^.

### ***In vivo*** administration of anti-γδ TCR

B10.RIII mice were injected intraperitoneally with 200 μg of anti-mouse γδ TCR Abs every 5 days (clone UC7-13D5; BioLegend). Control mice received equal amounts of armenian hamster IgG isotype control antibodies. Anti-γδ TCR efficacy was tested by FACS analysis of CD3^+^ TCRδ^+^ in the spleen and lymph nodes (Supplemental Fig. 1A).

### Clinical and Histological Methods

Disease severity for each limb is recorded as follows: 0 = normal; 1 = erythema and swelling of one digit; 2 = erythema and swelling of two digits 3 = erythema and swelling of more than two digits and/or swelling ankle joint. The clinical arthritis score was defined as the sum of the scores for all four paws of each mouse. Incidence was expressed as the percentage of mice with a disease score ≥1. Whole-ankle joints were fixed in 10% formalin decalcified in 10% EDTA and embedded in paraffin. Serial sections (4 μm) were stained with haematoxylin and eosin. Positive identification of neutrophils into the arthritic joints was determined by nuclear morphology and cytoplasmic color. The slides were obtained by Olympus BX61 confocal microscope and were analyzed with cellSens Dimension software.

### Flow cytometry

Blood samples were collected in EDTA tubes (Sarstedt) from tail bleeds, were labeled with leucocyte-specific antibodies as previously described^47^. Spleen was enzymatically digested for 30 min at 37 °C in Hank’s balanced salt solution (Life Technologies) containing 1 mg/ml collagenase D (Sigma) and pushed through a 70-μm cell strainer to obtain a single-cell suspension, which was then stimulated and/or stained. BM was flushed out of femur by use of a 27-gauge needle attached to a 10 ml syringe filled with PBS. Red blood cells were lysed with BD Pharm Lyse (BD Biosciences). Non-specific binding was blocked with TruStain FcX antibody (BioLegend) for 10 min at 4 °C in FACS buffer (Ca^2+^/Mg^2+^-free PBS with 2% FBS and 0.5 M EDTA) before staining (30 min) with appropriate antibodies. Abs were purchased from BioLegend and included.

CD45 (30-F11), CD11b (M1/70), Ly-6C (HK1.4), Ly-6G (1A8) CD115 (AFS98), class II major histocompatibility complex (MHC) (IA/IE), CD11c (N418), CD64 (X54-5/7.1), TCRγδ (GL3), CD3ε (145-2C11). Isotype controls Abs were used at the same protein concentrations as their corresponding markers. As a gating strategy we used forward- and side-scatter parameters to exclude cell aggregates and debris from analysis. Cells and beads (polyscience) were counted on an Attune Cytometer (Life Technologies) and analyzed using FlowJo software (Tree Star, Ashland, OR, USA).

### Cell sorting

For analysis of IL-27p28 mRNA, splenocytes from 12-weeks-old mice γδ TCR^+/+^ and γδ TCR^−/−^ mice were stimulated for 5 H with 1 μg/mL LPS. Then, macrophages (CD64^+^ CD11b^int^), dendritic cells (CD11c^hi^, MHCII^+^) and neutrophils (CD11b^+^ Ly-6G^+^) were sorted for qPCR analysis (Supplementary Fig. 2A). Dead cells and debris were excluded from the analysis using Zombie NIR™ Fixable Viability kit (Biolegend). Sorting was performed on a FACSAria II (BD Biosciences) and was reliably >90% of target population.

### Quantitative real-time RT-PCR

Total RNA from sorted cells was prepared using RNeasy Mini Kit (QIAGEN). cDNA was prepared using iScript cDNA Synthesis Kit (Bio-Rad, Hercules, CA). Quantitative real-time PCR was performed using iTaq Universal SYBR Green Supermix (Bio-Rad, Hercules, CA) according to the manufacturer’s instructions in a final volume of 20 μL, starting with a 5 min template denaturation step at 95 °C followed by 40 cycles of 15 s at 95 °C and 1 min at 60 °C with the following primers: Mouse *Gapdh* 5′-*TGGCCTTCCGTGTTCCTAC*-3′ and 5′-GAGTTGCTGTTGAAGTCGCA-3′ *Il-27p28* 5′-*CAGGATTCAAATGTTCAAAGG*-3′ and 5′-GGGCAGCTTCTTTTCTTCTT-3′. Relative expression of real-time PCR products was determined by using the ΔΔCt method to compare target gene and *GAPDH* mRNA expression.

### Chemotaxis experiment

Chemotactic migration of sorted blood neutrophils toward 10 ng/ml of IL-23 and IL-27 (from R&D Systems) or culture medium (negative control) was tested using IBIDI u slide chemotaxis. Cells (0.3 × 10^6^ cells in 6 μ) were loaded into the central transversal chamber and incubated at 37 °C for 60 minutes to allow cell attachment. Fresh RPMI was loaded into adjacent reservoirs and a chemotactic gradient was created following the manufacturer’s instructions. Neutrophil migration was monitored by analyzing captured images in 2 min intervals for a total duration of 2 hours with 40X objective using Keyence BZ-9000 microscope. Images were analyzed with the ImageJ software using the Manual Tracking plugin. Chemotaxis plots and migration parameters (distances and velocities) were obtained with the Chemotaxis and Migration tool from Ibidi.

### Statistical analysis

All results are expressed as mean ± SEM. Unpaired Student’s *t* test was used for determination of the significance of differences between two groups. One-way ANOVA was used to determine statistical significances in groups larger than two. A probability value of less than 0.05 is considered significant. Statistical analyses were performed using GraphPad Prism VI software (GraphPad Software, Inc.).

## Electronic supplementary material

Supplemental Figures
