# Supplementary material for: γδTCR regulates production of interleukin-27 by neutrophils and attenuates inflammatory arthritis
Source: Sci Rep. 2018 May 15;8:7590. doi: 10.1038/s41598-018-25988-3 (PMC5954154; doi:10.1038/s41598-018-25988-3)
Supplement: Supplementary file 1 — Supplemental Figures [file 41598_2018_25988_MOESM1_ESM.pdf]

γδTCR regulates production of interleukin-27 by neutrophils and attenuates inflammatory arthritis

Laura Bouchareychas*, Eva M Grössinger*, Mincheol Kang *, Iannis E Adamopoulos*†‡

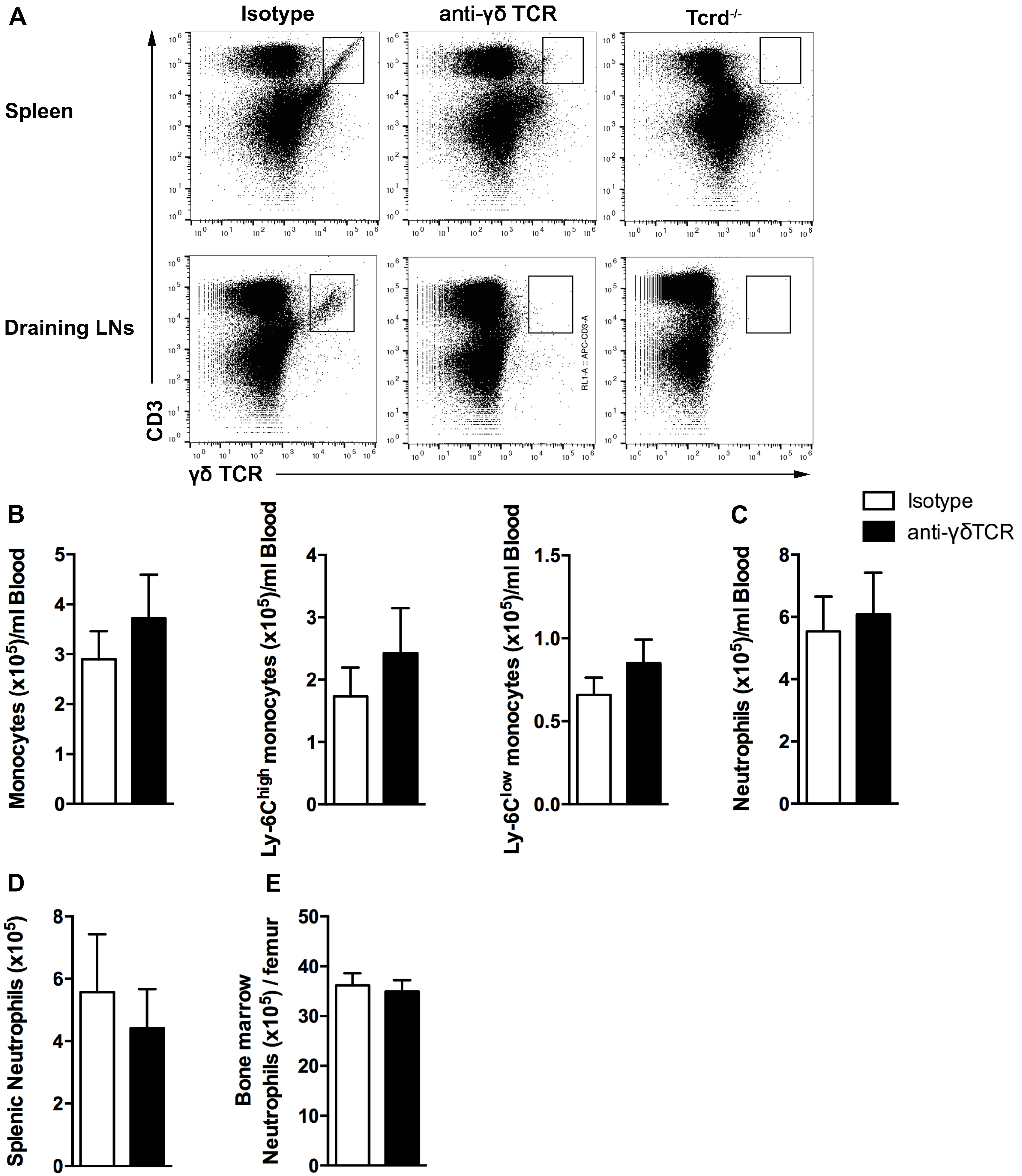

Supplemental Figure 1. A) Representative dot plots of γδ T cells gated on single cells in the spleen and draining lymph nodes (LNs) of B10.RIII mice treated with isotype or anti-γδTCR mAb compared to γδTCR$^{-/-}$ mice.

B) Quantification by flow cytometry analysis of of total blood monocytes, Ly-6C ^{high} and Ly-6C ^{low} subsets (**C**) Quantification of blood neutrophils (**D**) splenic neutrophils and (**E**) bone marrow neutrophils in B10.RIII mice treated with anti-γδ TCR mAb or isotype. Data are representative of three experiments with 5 or 6 mice per group.

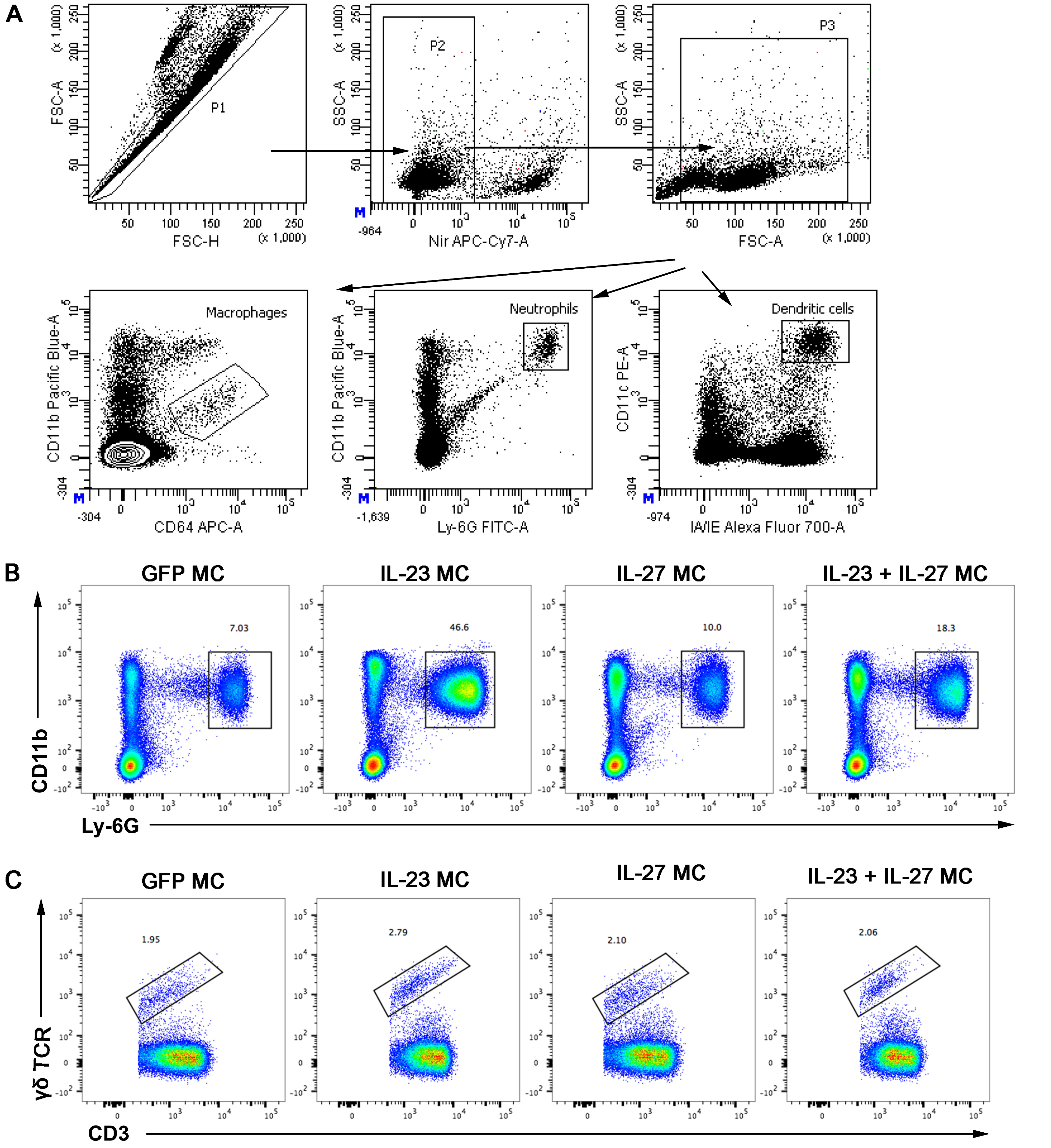

Supplemental Figure 2A. Gating strategy used to obtain macrophages, neutrophils and dendritic cell populations as shown with one representative splenocytes staining. After exclusion of doublets (P1), dead cells (P2), cell aggregates and debris (P3), we selected macrophages as $CD64^+$ $CD11b^{int}$ cells, neutrophils asm$CD11b^+$ Ly-$6G^+$ cells., dendritic cells as $CD11c^{hi}$ $MHCII^+$ cells. **B**) Representative dot plot profiles showing the gating strategy that was used to identify blood neutrophils in B10.RIII mice injected with GFP, IL-23 MC, IL-27 MC or both IL-23 + IL-27 MC at day 11. Numbers depict the percentage of $CD45^+$ leukocytes. **C**) Representative dot plots of γδTCR gated on $CD3^+$ cells in the spleen of B10.RIII mice injected with GFP, IL-23 MC, IL-27 MC or both IL-23 + IL-27 MC at day 11. Numbers depict the percentage of $CD3^+$ cells. Data are representative of two independent experiments.
